# A case of amniotic fluid embolism successfully treated by multidisciplinary treatment

**DOI:** 10.1186/s40981-019-0296-0

**Published:** 2019-11-28

**Authors:** Yuki Kinishi, Chiyo Ootaki, Takeshi Iritakenishi, Yuji Fujino

**Affiliations:** Department of Anesthesiology and Intensive Care, Osaka University Graduate School of Medicine, 2-2 Yamadaoka, Suita, Osaka, 565-0871 Japan

**Keywords:** Labor, Epidural, Advanced maternal age

## Abstract

**Background:**

Amniotic fluid embolism (AFE) is a life-threatening obstetric emergency. Because the maternal mortality associated with AFE is very high, early recognition and prompt treatment are important for improving the prognosis. We report a case of amniotic fluid embolism successfully treated by multidisciplinary treatment.

**Case presentation:**

A 39-year-old woman with fetal congenital heart anomaly and polyhydramnios was scheduled for induction of delivery at 37 weeks of gestation with labor epidural analgesia. Uncontrollable bleeding occurred 30 min after vaginal delivery. Based on the clinical diagnosis of AFE, massive blood transfusion, insertion of an aortic occlusion balloon catheter, and hysterectomy was performed. Total blood loss was 12,000 mL. The diagnosis of AFE was confirmed by pathological examination. She was discharged with no complications.

**Conclusion:**

We report a case of AFE who were rescued by prompt diagnosis and treatment.

## Background

Amniotic fluid embolism (AFE) is known as a serious reaction triggered by amniotic fluid or by other debris entered in the maternal circulation. It has been recently suggested that amniotic fluid embolism is an anaphylactic syndrome of pregnancy involving the complement system, causing vasospasm, edema, and early onset disseminated intravascular coagulation (DIC), which is one of the causes of sudden death in obstetrics [1–3]. The mortality of AFE is very high, varying from 20 to 60% [3]. In Japan, 93 maternal death cases were autopsied from 1989 to 2005, of which AFE was the most common cause of death (24.3%) [4]. Early recognition and prompt resuscitation are the key factors for the treatment of AFE. We report a case of amniotic fluid embolism successfully treated by multidisciplinary treatment.

## Case presentation

A 39-year-old, gravida 2, para 1, Japanese woman (158 cm/56.8 kg) with fetal congenital heart anomaly and polyhydramnios was scheduled for induction of delivery at 37 weeks of gestation. She requested labor analgesia.

The combined spinal-epidural block was placed at L3-L4 level, and 1.5 mg 0.5% isobaric bupivacaine with fentanyl 15 mcg was administered into the subarachnoid space. A catheter was introduced into the epidural space at cervix dilatation of 4 cm. Then, the labor analgesia was managed by programmed intermittent epidural bolus with 0.1% ropivacaine and fentanyl 2 mcg/mL was set at 8 mL with a 60-min interval. Due to polyhydramnios, pinhole amniotomy was performed at cervix dilation of 3 cm. There were no major complications until fetal bradycardia (80–90 beats per minute) occurred, which prompted the obstetric physician to go for vacuum extraction delivery. Patient delivered a female infant weighing 2468 g with an Apgar score of 7/8. Total delivery time was 2 h and 39 min (second stage of labor duration was 22 min).

**Fig. 1:**
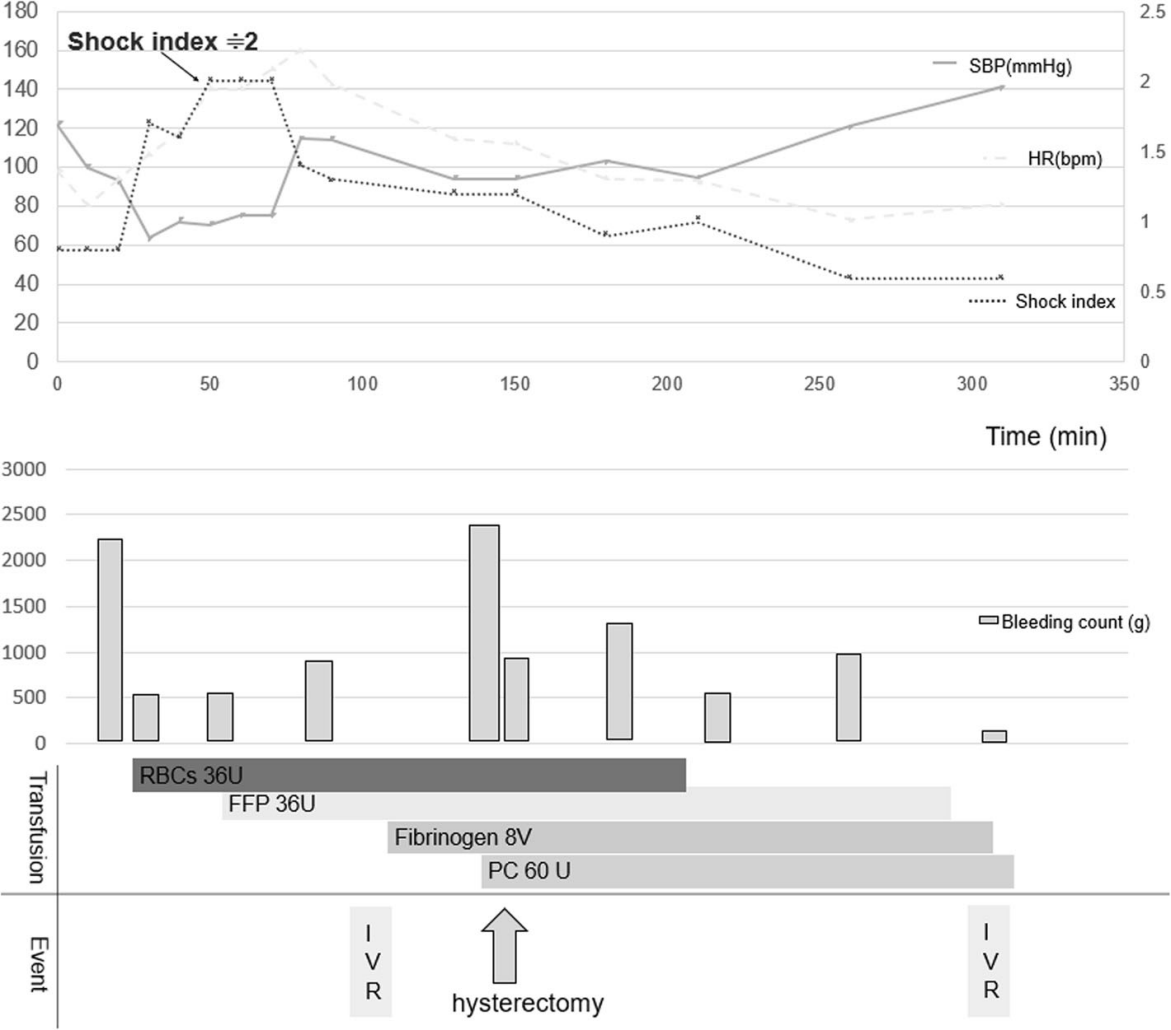
The perioperative chart from 0 to 350 min. Time 0 was defined as the time the fetus was delivered. The interventional radiology (IVR) and a total hysterectomy were performed to control bleeding. Red blood concentrates (RBCs) and fresh frozen plasma (FFP) were transfused using a rapid infusion system. After hysterectomy, the fibrinogen level and blood pressure were increased, the shock index was decreased. However, the second IVR was required to control bleeding from the vagina. At last, her bleeding was controlled

After an episiotomy, a large amount of bleeding from the uterus was observed, and the obstetric physician suspected it as postpartum atony. Blood pressure was 112/89 mmHg, heart rate was 80 beats per minute, shock index was 1, and total amount of bleeding was 2800 mL at that time. We started to transfuse red blood cells and placed an intrauterine (Bakri®) balloon. At 30 min after the delivery, the total bleeding amount reached 3100 mL, and the blood pressure was decreased to 72/43 mmHg, shock index increased to 2, and SpO_2_ decreased to 86%. We started treatment based on a suspicious diagnosis of AFE due to sudden decrease of plasma fibrinogen level and uncontrollable bleeding from a needle hole. She was orotracheally intubated, and a central venous and a radial artery catheter was inserted. We ruled out pulmonary embolism because the central venous pressure was 5 mmHg. Besides administering low-dose noradrenaline, an occlusion balloon was inserted into the descending aorta. Uterine artery embolization was performed at radiology department. Red blood concentrates (RBCs) and fresh frozen plasma (FFP) were transfused using a rapid infusion system. In spite of those treatments, her bleeding was uncontrollable and obstetrics team decided to perform total hysterectomy, and the patient was transferred to the operating room. The time from the start of surgery to hysterectomy was 11 min. The fibrinogen level increased above 100 mg/dL after hysterectomy. However, the second interventional radiology (IVR) was required to control bleeding from the vagina. Embolization of the right internal pudendal artery and cervicovaginal branches of the right uterine artery was performed. At last, her bleeding was controlled, and the blood pressure became stable without norepinephrine administration. After hysterectomy, she was transferred to the ICU with intubation. The total bleeding amount was 12,000 mL, and the total amount of RBCs, FFP, platelet concentrate, and fibrinogen required were 38 U, 36 U, 60 U, and 8 V, respectively. The perioperative chart is shown in Fig. 1. The time series results of blood sampling are shown in Table 1.

**Table 1:** Laboratory data

	Postpartum bleeding begins	Before ER	After 1st IVR	After hysterectomy	One hour after hysterectomy	At OR	After 2nd IVR
Fib (mg/dL) [200–400]	25>	25>	25>	104	122	251	246
WBC (× 10^3^/μL) [30–80]	21.28	19.56	19.85	9.36	7.69	8.32	8.31
Hb (g/dL) [11–15]	9.5	10.5	10.4	7.3	8.7	10.1	9.0
Plt (× 10^3^/μL) [150–360]	123	107	120	80	114	78	136

*Fib* fibrinogen, *WBC* white blood count, *Hb* hemoglobin, *Plt* platelet count, *ER* emergency room, *IVR* interventional radiology, *OR* operating room

She was extubated on the second day after surgery at ICU. On the third day, she was moved from the ICU to the general ward. On the 16th postoperative day, she was discharged from the hospital without any complications. Laboratory tests at when postpartum hemorrhage began showed that serum zinc coproporphyrin-1 and sialyl-Tn antigens were negative, but C3 (64.0 mg/dL), C4 component (10.0 mg/dL), and C1 esterase inhibitor levels (27%) were low. A month later, immunostaining examination revealed fetal components in the uterus, confirming a diagnosis of DIC type AFE.

## Discussion

AFE in our case was successfully rescued due to two factors: (1) early diagnosis and (2) early intervention by the medical team. AFE can be divided into two clinical types: (1) cardiopulmonary collapse type and (2) DIC type involving atonic bleeding and rapid deterioration of coagulopathy [1]. The differential diagnosis of DIC type AFE in our case from normal obstetric DIC was made based on a rapid decrease of fibrinogen. In DIC type AFE, the influx of blood coagulation-promoting substances in the uterus causes rapid activation of extrinsic coagulation pathways, and fibrinogen drastically reduces. On the other hand, in normal obstetric DIC, fibrinogen decreases according to the massive bleeding and the DIC is caused secondarily by the diluted coagulopathy [5]. Since the blood coagulation factor is depleted early by the anaphylactoid reaction, it is important to supply the coagulation factor by administering large amounts of FFP from the early stage for DIC type AFE [1, 6].

DIC type AFE has been reported to cause cardiac arrest in approximately 60% cases within 6 h after the appearance of symptoms, and as in this case, early intervention is important [7]. Therefore, it is necessary to transport a patient to a larger medical facility in case of obstetric crisis [8]. Pregnant women with labor epidural analgesia have a lower risk of critical postpartum hemorrhage compared with those without it, probably because immediate examination and management of postpartum hemorrhage is facilitated by an epidural catheter [9]. Fortunately, this situation, including the fact that she requested parturition epidural analgesia, made possible her resuscitation by the medical team, which included anesthesiologists, who possessed the patient’s information before the occurrence of AFE.

A diagnosis of AFE is made by autopsy and clinical diagnostic criteria, and serologic tests are also used for assisted diagnosis [1]. Serum markers are available for making an auxiliary diagnosis of AFE. In this case, the C3 and C4 complement and C1 esterase inhibitor were low. The serum levels of C3 and C4 complement had a high sensitivity and specificity for the diagnosis of AFE [10]. The C1 esterase inhibitor decreased in patients with AFE [11].

This patient had a risk of AFE due to advanced maternal age, polyhydramnios, amniotomy, and induction of delivery [1, 12, 13]. Patients with advanced maternal age are at risk of AFE and thus should be managed attentively. Due to social background and progress of infertility treatment, it is expected that cases of advanced maternal age would increase in the future. It may be necessary to improve the medical system so that intensification of delivery facilities or close collaboration between clinics and higher medical facilities can be made in Japan.

## Conclusions

We report a case of AFE in advanced maternal age with labor epidural anesthesia. The medical team and anesthesiologists had a large role in the maternal life-saving condition.

## Data Availability

Please contact the corresponding author for data requests.

## Abbreviations

AFE: Amniotic fluid embolism
C: Complement
DIC: Disseminated intravascular coagulation
FFP: Fresh frozen plasma
HR: Heart rate
IVR: Interventional radiology
PC: Platelet concentrate
RBCs: Red blood concentrates
SBP: Systolic blood pressure
Shock index: The heart rate divided by the systolic blood pressure
